# Supplementary material for: Colletotrichum Species Associated with Peaches in China
Source: J Fungi (Basel). 2022 Mar 18;8(3):313. doi: 10.3390/jof8030313 (PMC8950351; doi:10.3390/jof8030313)
Supplement: Supplementary file 1 [file jof-08-00313-s001.zip › jof-1629201-Supplementary.pdf]

Table S1. Primers used in this study, with sequences, annealing temperature and sources.

Gene/Locus	Primer name	Direction	Primer sequence (5'to 3')	Ta(℃)	Reference
ITS	ITS1-F	Forward	CTTGGTCATTTAGAGGAAGTAA	52	Gardes and Bruns [78]
	ITS-4	Reverse	TCCTCCGCTTATTGATATGC		White et al. [79]
GAPDH	GDF1	Forward	GCCGTCAACGACCCCTTCATTGA	60	Guerber et al. [80]
	GDR1	Reverse	GGGTGGAGTCGTACTTGAGCATGT		
CHS-1	CHS-79F	Forward	TGGGGCAAGGATGCTTGGAAGAAG	58	Carbone and Kohn [81]
	CHS-354R	Reverse	TGGAAGAACCATCTGTGAGAGTTG		
ACT	ACT-512F	Forward	ATGTGCAAGGCCGGTTTCGC	58	Carbone and Kohn [81]
	ACT-783R	Reverse	TACGAGTCCTTCTGGCCCAT		
TUB2	Btub2Fd	Forward	GTBCACCTYCARACCGGYCARTG	52	Woudenberg et al. [82]
	Btub4Rd	Reverse	CCRGAYTGRCCRAARACRAAGTTGTC		
	T1	Forward	AACATGCGTGAGATTGTAAGT	52	O'Donnell and Cigelnik [83]
	Bt2b	Reverse	ACCCTCAGTGTAGTGACCCTTGGC		Glass and Donaldson [84]
HIS3	CYLH3F	Forward	AGGTCCACTGGTGGCAAG	52	Crous et al. [85]
	CYLH3R	Reverse	AGCTGGATGTCCTTGGACTG		
CAL	CL1C	Forward	GAATTCAAGGAGGCCTTCTC	59	Weir et al. [51]
	CL2C	Reverse	CTTCTGCATCATGAGCTGGAC		

Table S2. Isolates of seven *Colletotrichum* species collected from peaches in China, with details about host tissue, location, and GenBank accession number.

Species	Isolate [a]	Host tissue	Location	GenBank accession number						
				ITS	*GAPDH*	*CHS-1*	*ACT*	*TUB2*	*HIS3*	*CAL*
C. fioriniae	ZJLS 1	Fruit	Lishui, Zhejiang	MN807705	MN829566	MN829685	MN829616	MN829766	MN829735	-
	ZJLS 6-2	Fruit	Lishui, Zhejiang	MN807706	MN829567	MN829686	MN829617	MN829767	MN829736	-
	ZJLS 11-1	Fruit	Lishui, Zhejiang	MN807707	MN829568	MN829687	MN829618	MN829768	MN829737	-
	GZTR 4-1	Fruit	Tongren, Guizhou	MN807708	MN829569	MN829688	MN829619	MN829769	MN829738	-
	GZTR 7-1	Fruit	Tongren, Guizhou	MN807709	MN829570	MN829689	MN829620	MN829770	MN829739	-
	JXJA 1	Fruit	Jian, Jiangxi	MN807710	MN829571	MN829690	MN829621	MN829771	MN829740	-
	JXJA 3	Fruit	Jian, Jiangxi	MN807711	MN829572	MN829691	MN829622	MN829772	MN829741	-
	JXJA 6	Fruit	Jian, Jiangxi	MN807712	MN829573	MN829692	MN829623	MN829773	MN829742	-
C. folicola	YNHH 2-2	Leaf	Honghe,Yunnan	MN807724	MN829585	MN829704	MN829635	MN829785	MN829754	-
	YNHH 10-1, CCTCC M 2020345*	Leaf	Honghe,Yunnan	MN807725	MN829586	MN829705	MN829636	MN829786	MN829755	-
C. fructicola	GZTR 10-1	Fruit	Tongren,Guizhou	MN807726	MN829587	MN829706	MN829637	MN829787	-	MN829656
	GZTR 11-1	Fruit	Tongren,Guizhou	MN807727	MN829588	MN829707	MN829638	MN829788	-	MN829657
	GZTR 13-1	Fruit	Tongren,Guizhou	MN807728	MN829589	MN829708	MN829639	MN829789	-	MN829658
	GDHY 1	Fruit	Heyuan,Guangdong	MN807729	MN829590	MN829709	MN829640	MN829790	-	MN829659
	GDHY 5-2	Fruit	Heyuan,Guangdong	MN807730	MN829591	MN829710	MN829641	MN829791	-	MN829660
	GDHY 6-1	Fruit	Heyuan,Guangdong	MN807731	MN829592	MN829711	MN829642	MN829792	-	MN829661
	GDHY 10-1	Fruit	Heyuan,Guangdong	MN807732	MN829593	MN829712	MN829643	MN829793	-	MN829662
	GDSG 1-1	Fruit	Shaoguan,Guangdong	MN807733	MN829594	MN829713	MN829644	MN829794	-	MN829663
	GDSG 3-1	Fruit	Shaoguan,Guangdong	MN807734	MN829595	MN829714	MN829645	MN829795	-	MN829664
	GDSG 5-1	Fruit	Shaoguan,Guangdong	MN807735	MN829596	MN829715	MN829646	MN829796	-	MN829665
C. godetiae	YNHH 1-1	Leaf	Honghe,Yunnan	MN807713	MN829574	MN829693	MN829624	MN829774	MN829743	-
	YNHH 2-1	Leaf	Honghe,Yunnan	MN807714	MN829575	MN829694	MN829625	MN829775	MN829744	-

	YNHH 4-1	Leaf	Honghe,Yunnan	MN807715	MN829576	MN829695	MN829626	MN829776	MN829745	-
	YNHH 6-1	Leaf	Honghe,Yunnan	MN807716	MN829577	MN829696	MN829627	MN829777	MN829746	-
	YNHH 7-2	Leaf	Honghe,Yunnan	MN807717	MN829578	MN829697	MN829628	MN829778	MN829747	-
	YNHH 8-1	Leaf	Honghe,Yunnan	MN807718	MN829579	MN829698	MN829629	MN829779	MN829748	-
	YNHH 9-1	Leaf	Honghe,Yunnan	MN807719	MN829580	MN829699	MN829630	MN829780	MN829749	-
	YNHH 11-1	Leaf	Honghe,Yunnan	MN807720	MN829581	MN829700	MN829631	MN829781	MN829750	-
C. karsti	YNHH 3-1	Leaf	Honghe,Yunnan	MN807721	MN829582	MN829701	MN829632	MN829782	MN829751	MT732370
	YNHH 3-2	Leaf	Honghe,Yunnan	MN807722	MN829583	MN829702	MN829633	MN829783	MN829752	MT732371
	YNHH 5-2	Leaf	Honghe,Yunnan	MN807723	MN829584	MN829703	MN829634	MN829784	MN829753	MT732372
C. nymphaeae	HBYC 1	Fruit	Yichang,Hubei	MN807695	MN829556	MN829675	MN829606	MN829756	MN829725	
	HBJM 1-1	Fruit	Jingmen,Hubei	MN807696	MN829557	MN829676	MN829607	MN829757	MN829726	-
	HBWH 2-1	Fruit	Wuhan,Hubei	MN807697	MN829558	MN829677	MN829608	MN829758	MN829727	-
	HBWH 3-2	Leaf	Wuhan,Hubei	MW157938	MW161235	MW161236	MW161237	MW161238	MW161239	
	HBXG 1	Fruit	Xiaogan,Hubei	MN807698	MN829559	MN829678	MN829609	MN829759	MN829728	-
	SCCD 1	Fruit	Chengdu,Sichuan	MN807699	MN829560	MN829679	MN829610	MN829760	MN829729	-
	GZQZ 4	Fruit	Qingzhen,Guizhou	MN807700	MN829561	MN829680	MN829611	MN829761	MN829730	-
	GXGL 3-1	Fruit	Guilin,Guangxi	MN807701	MN829562	MN829681	MN829612	MN829762	MN829731	-
	GXGL 12-1	Leaf	Guilin,Guangxi	MN807702	MN829563	MN829682	MN829613	MN829763	MN829732	-
	FJFZ 1	Fruit	Fuzhou,Fujian	MN807703	MN829564	MN829683	MN829614	MN829764	MN829733	-
	GZTR 8-1	Fruit	Tongren,Guizhou	MN807704	MN829565	MN829684	MN829615	MN829765	MN829734	-
C. siamense	SDQD 1-1	Fruit	Qingdao,Shandong	MN807736	MN829597	MN829716	MN829647	MN829797	-	MN829666
	SDQD 5	Fruit	Qingdao,Shandong	MN807737	MN829598	MN829717	MN829648	MN829798	-	MN829667
	SDQD 7-1	Fruit	Qingdao,Shandong	MN807738	MN829599	MN829718	MN829649	MN829799	-	MN829668
	SDQD 10-1	Fruit	Qingdao,Shandong	MN807739	MN829600	MN829719	MN829650	MN829800	-	MN829669
	SDQD 15-2	Fruit	Qingdao,Shandong	MN807740	MN829601	MN829720	MN829651	MN829801	-	MN829670
	HBSJZ 1-1	Fruit	Shijiazhuang,Hebei	MN807741	MN829602	MN829721	MN829652	MN829802	-	MN829671

HBSJZ 3-1	Fruit	Shijiazhuang,Hebei	MN807742	MN829603	MN829722	MN829653	MN829803	-	MN829672
HBSJZ 5-1	Fruit	Shijiazhuang,Hebei	MN807743	MN829604	MN829723	MN829654	MN829804	-	MN829673
HBSJZ 7-1	Fruit	Shijiazhuang,Hebei	MN807744	MN829605	MN829724	MN829655	MN829805	-	MN829674

[a] CCTCC: China Center for Type Culture Collection. * = Ex-type culture.

Table S3. The sizes of conidia, appresoria, ascospores and mycelial growth rate of the representative isolates of *Colletotrichum* spp. obtained in this study.

**Species and Isolate**	**Conidia** [a]			**Appresoria** [b]			**Ascospores** [c]			**Mycelial growth rate (mm/d)** [d]	
	**Length (μm)**	**Width (μm)**	**Means ± SD of conidia (μm)**	**Length (μm)**	**Width (μm)**	**Means ± SD of appresoria (μm)**	**Length (μm)**	**Width (μm)**	**Means ± SD of ascospores (μm)**	**25℃**	**30℃**
C. nymphaeae	8.8-16.6	3.1-6.3	12.1±0.9a×4.6±0.5a	4.1-12.8	2.2-6.7	7.2±0.5a×4.9±0.4a	/	/	/	6.3±0.2a	3.7±0.3bc
FJFZ 1	9.2-13.0	3.6-5.2	11.5±1.0×4.3±0.4	5.5-9.3	3.5-5.6	6.9±0.8×4.9±0.5	/	/	/	6.5±0.2	3.7±0.2
GXGL 13-1	11.5-16.6	3.9-6.2	14.0±1.3×5.0±0.5	5.6-10.3	4.2-5.8	7.3±1.0×5.1±0.4	/	/	/	6.7±0.6	4.3±0.2
GZTR 8-1	9.1-12.9	3.7-5.1	11.4±1.0×4.4±0.4	4.1-12.8	2.2-6.0	8.3±2.0×4.5±0.9	/	/	/	6.1±0.4	3.2±0.3
HBJM 1-1	8.8-13.0	3.5-5.1	11.4±1.1×4.5±0.3	5.1-12.3	2.7-5.7	7.5±1.6×4.2±0.7	/	/	/	6.3±0.4	3.5±0.2
HBWH 2-1	11.1-14.4	3.9-5.2	12.5±0.9×4.5±0.3	4.9-9.4	4.3-6.5	6.8±1.0×5.2±0.5	/	/	/	6.4±0.5	3.5±0.3

HBWH 3-2	9.9-13.3	4.4-6.3	11.9±0.8×5.1±0.4	5.0-8.6	4.1-6.2	6.5±0.8×5.2±0.5	/	/	/	6.0±0.2	3.5±0.2
HBXG 1	9.2-13.8	3.6-4.8	11.9±1.1×4.3±0.3	5.3-10.9	3.4-5.6	7.5±1.6×4.6±0.5	/	/	/	6.4±0.2	4.1±0.3
HBYC 1	10.4-14.8	4.7-6.1	12.9±0.9×5.4±0.4	6.1-9.4	4.2-6.7	7.3±0.9×5.5±0.6	/	/	/	6.4±0.6	3.6±0.3
SCCD 1	8.9-13.2	3.1-5.0	11.4±1.1×4.1±0.4	5.7-8.7	4.3-6.5	7.1±0.9×5.4±0.4	/	/	/	6.3±0.5	3.8±0.4
C. fioriniae	11.5-17.2	3.6-6.4	14.2±0.6bc×5.0±0.5ab	5.0-11.6	3.7-6.9	7.1±0.5a×5.2±0.2ab	/	/	/	7.5±0.5a	4.7±0.3c
GZTR 7-1	12.3-16.9	4.2-5.2	14.1±1.1×4.6±0.3	6.4-11.6	4.3-6.9	7.5±1.0×5.4±0.5	/	/	/	8.0±0.4	5.1±0.2
JXJA 1	11.5-15.9	3.6-5.3	13.8±1.0×4.7±0.3	6.2-10.2	3.7-6.6	7.5±1.1×5.0±0.7	/	/	/	7.9±0.2	4.5±0.1
JXJA 6	12.3-17.2	5.3-6.4	15.2±1.2×5.8±0.3	5.9-8.3	4.1-6.4	7.1±0.6×5.3±0.5	/	/	/	7.4±0.4	4.8±0.5
ZJLS 1	11.6-17.1	3.7-5.1	14.1±1.3×4.7±0.3	5.0-7.4	3.7-6.1	6.2±0.7×5.0±0.6	/	/	/	7.0±0.4	4.8±0.4
ZJLS 11-1	12.0-15.7	4.5-6.2	13.7±0.8×5.2±0.5	5.6-9.9	4.3-6.4	7.3±0.9×5.3±0.5	/	/	/	7.0±0.3	4.3±0.4
C. godetiae	10.7-17.5	4.2-6.8	14.4±0.5cd×5.4±0.4b	6.2-14.9	3.3-9.5	9.3±1.0c×5.4±0.8ab	/	/	/	6.7±1.4a	1.0±0.1a
YNHH 1-1	12.6-16.5	4.2-5.3	14.0±0.8×4.8±0.3	6.2-13.4	4.1-6.4	8.5±1.6×5.0±0.5	/	/	/	7.4±0.1	1.0±0.5
YNHH 4-1	12.0-15.5	4.7-6.5	14.1±0.9×5.4±0.4	7.7-14.2	4.1-6.6	10.3±1.5×5.4±0.6	/	/	/	7.4±0.2	1.0±0.4
YNHH 6-1	12.7-17.5	4.7-6.8	15.3±1.3×5.7±0.6	6.3-12.7	3.3-6.6	9.5±1.8×5.0±0.8	/	/	/	7.7±0.3	1.1±0.5
YNHH 8-2	10.7-15.7	4.5-6.0	14.2±1.2×5.2±0.3	6.9-14.9	5.0-9.5	10.6±2.0×6.9±1.1	/	/	/	6.4±0.3	0.9±0.5
YNHH 9-1	12.1-15.4	4.6-6.0	13.9±0.8×5.2±0.4	6.5-9.3	4.6-7.0	8.1±0.6×5.5±0.5	/	/	/	7.6±0.1	1.1±0.5
C. fructicola	9.3-18.9	3.4-8.2	14.3±1.7cd×5.6±0.5b	6.2-10.6	4.5-7.3	8.0±0.6ab×5.9±0.5bc	12.6-22.0	3.1-7.6	17.3±0.5a×5.0±0.5a	11.1±1.1c	10.8±1.2e
GDHY 10-1	13.8-18.9	4.7-8.2	16.4±1.2×6.2±0.8	6.8-10.3	5.0-6.9	8.6±0.7×5.9±0.6	12.8-22.0	3.1-6.5	17.3±2.4×5.1±0.8	9.5±0.3	9.2±0.3
GDSG 1-1	13.8-17.2	4.8-5.9	14.9±0.8×5.5±0.3	7.2-10.6	5.6-7.3	8.2±0.8×6.4±0.4	13.5-20.4	3.4-6.7	16.8±2.0×5.0±0.8	11.3±0.3	11.2±0.3
GDSG 5-1	12.1-15.8	4.2-6.5	13.8±1.0×5.5±0.5	6.8-8.5	5.2-7.0	7.6±0.4×6.0±0.4	14.9-19.9	3.6-7.6	17.9±1.3×5.5±0.7	11.3±0.1	10.8±0.4
GZTR 10-1	9.3-15.8	3.4-6.1	12.3±1.5×5.1±0.6	6.2-8.5	45-6.4	7.3±0.6×5.3±0.5	12.6-21.7	3.4-5.8	17.0±2.2×4.4±0.6	12.1±0.4	11.9±0.2

C. siamense	13.2-18.3	4.6-6.3	15.3±0.4d×5.4±0.3b	6.3-9.8	4.5-7.3	8.0±0.2ab×5.8±0.4bc	/	/	/	11.2±0.4c	11.1±1.8e
SDQD 1-1	14.0-17.5	4.6-6.0	15.6±1.0×5.2±0.3	7.2-9.5	4.7-6.9	8.0±0.6×5.7±0.5	/	/	/	10.8±0.2	8.5±0.6
SDQD 10-1	13.2-18.3	5.0-6.1	15.7±1.1×5.6±0.3	6.7-8.9	5.4-7.2	8.0±0.5×6.4±0.5	/	/	/	11.8±0.3	12.3±0.6
HBSJZ 1-1	13.2-16.6	4.8-6.3	14.8±1.0×5.6±0.4	6.4-9.8	4.5-7.3	7.7±0.9×5.6±0.6	/	/	/	11.2±0.8	12.0±0.5
HBSJZ 3-1	13.2-18.3	4.6-5.8	15.2±1.3×5.1±0.4	6.3-9.5	4.7-7.0	8.1±0.9×5.4±0.5	/	/	/	11.1±1.0	11.8±0.8
C. karsti	10.6-14.9	5.8-7.4	12.9±0.3ab×6.7±0.2c	6.8-14.4	5.1-7.8	9.3±0.8c×6.3±0.1c	11.5-17.8	4.0-7.1	14.6±0.6b×5.2±0.2a	8.9±0.1b	8.5±0.1d
YNHH 3-1	10.6-14.3	6.2-7.4	12.84±0.93×6.8±0.3	6.8-14.4	5.1-7.2	9.4±1.7×6.3±0.5	11.6-16.5	4.1-6.8	13.9±1.3×4.9±0.6	8.7±0.6	8.5±0.1
YNHH 3-2	11.4-14.9	6.3-7.4	13.3±0.8×6.7±0.3	7.2-13.0	5.1-7.6	10.0±1.4×6.4±0.6	11.5-17.8	4.0-7.1	14.9±1.5×5.3±0.8	8.8±0.5	8.7±0.3
YNHH 5-2	11.6-14.1	5.8-7.1	12.7±0.6×6.4±0.4	6.9-11.6	5.1-7.8	8.5±1.1×6.2±0.6	11.5-17.8	4.0-7.1	14.9±1.5×5.3±0.8	9.0±0.6	8.4±0.2
C. folicola	12.3-15.4	5.6-7.8	13.6±0.1bc×6.5±0.3c	5.6-13.7	4.0-8.2	8.4±0.5bc×5.9±0.1bc	/	/	/	6.3±0.4a	2.8±0.2b
YNHH 2-2	12.7-15.4	5.6-6.8	13.7±0.6×6.3±0.3	6.7-10.1	4.0-7.2	8.0±0.9×5.9±0.8	/	/	/	6.1±0.7	3.0±0.5
YNHH 10-1	12.3-15.0	5.6-7.8	13.5±0.7×6.7±0.6	5.6-13.7	4.7-8.2	8.7±2.5×5.8±1.0	/	/	/	6.6±0.6	2.7±0.4

[a] Minimum to maximum sizes for length and width of 30 conidia, and mean conidia size. Significance at P = 0.05 level.

[b] Minimum to maximum sizes for length and width of 30 appresoria, and mean appresoria size. Significance at P = 0.05 level.

[c] Minimum to maximum sizes for length and width of 30 ascospores, and mean ascospores size. Significance at P = 0.05 level.

[d] The colony diameter data were measured at the 3th days to calculate the mycelial growth rate (mm/day). Significance at P = 0.05 level.

/ Ascospores were absent.

Table S4. Infection rates of seven *Colletotrichum* spp. inoculated on peach fruit and leaves.

Species	Isolate	Origin	Infection rate on fruit	Infection rate on leaves
C. nymphaeae			60/60	52/60
	FJFZ 1	Fruit	12/12	10/12
	HBJM 1-1	Fruit	12/12	12/12
	HBWH 3-2	Leaf	12/12	12/12
	HBYC 1	Fruit	12/12	8/12
	SCCD 1	Fruit	12/12	10/12
C. fioriniae			60/60	56/60
	GZTR 7-1	Fruit	12/12	12/12
	JXJA 1	Fruit	12/12	10/12
	JXJA 6	Fruit	12/12	12/12
	ZJLS 1	Fruit	12/12	12/12
	ZJLS 11-1	Fruit	12/12	10/12
C. godetiae			54/60	53/60
	YNHH 1-1	Leaf	12/12	12/12
	YNHH 2-1	Leaf	8/12	8/12
	YNHH 4-1	Leaf	11/12	11/12
	YNHH 7-2	Leaf	12/12	11/12
	YNHH 9-1	Leaf	11/12	11/12
C. fructicola			60/60	60/60
	GDHY 10-1	Fruit	12/12	12/12
	GDSG 1-1	Fruit	12/12	12/12
	GDSG 5-1	Fruit	12/12	12/12
	GZTR 10-1	Fruit	12/12	12/12
	GZTR 13-1	Fruit	12/12	12/12
C. siamense			59/60	59/60
	HBSJZ 1-1	Fruit	12/12	12/12
	HBSJZ 3-1	Fruit	12/12	12/12
	HBSJZ 5-1	Fruit	12/12	11/12
	HBSJZ 7-1	Fruit	11/12	12/12
	SDQD 10-1	Fruit	12/12	12/12
C. karsti			7/36	23/36
	YNHH 3-1	Leaf	2/12	7/12
	YNHH 3-2	Leaf	3/12	9/12
	YNHH 5-2	Leaf	2/12	7/12
C. folicola			0/24	17/24
	YNHH 2-2	Leaf	0/12	10/12
	YNHH 10-1	Leaf	0/12	7/12
Control	H_2O		0/12	0/12
